# Access to medicines for diabetes treatment in Brazil: evaluation of “health has no price” program

**DOI:** 10.1186/s13098-016-0150-8

**Published:** 2016-05-10

**Authors:** João Leopoldo Oliveira Araujo, Mariana Donato Pereira, Cristiane de Cássia Bergamaschi, Fernando de Sá Del Fiol, Luciane Cruz Lopes, Maria Inês de Toledo, Silvio Barberato-Filho

**Affiliations:** Programa de Pós-graduação stricto sensu em Ciências Farmacêuticas, Universidade de Sorocaba, Rod Raposo Tavares, Km 92,5, Sorocaba, SP 18023-000 Brazil; Department of Pharmacy, University of Brasília, Brasília, DF Brazil

**Keywords:** Health policy, Health systems, Health services accessibility, Hypoglycemic agents, Diabetes mellitus

## Abstract

**Background:**

In 2011, private pharmacies associated to the Brazilian Ministry of Health provided patients with two types of insulin (regular human insulin and isophane insulin or NPH) and three oral antidiabetic medications (5 mg glibenclamide and 500 and 850 mg metformin) free of charge. The aim was to evaluate the impact of the “Health Has No Price” Program [*Saúde Não Tem Preço* (SNTP)] for access to diabetes treatment medicines in Brazil.

**Methods:**

This longitudinal and observational study is based on the number of units of oral hypoglycemic agents, insulin and insulin analogues supplied in 55,000 private pharmacies from February 1, 2010 to January 31, 2012. The number of tablets (oral hypoglycemic agents) and international units (insulins and insulin analogues) supplied in the first 12 months of the SNTP Program were compared with the number of tablets and international units supplied in the 12 months prior to its implementation.

**Results:**

The insulins in the SNTP program had the highest percentage change in the number of international units supplied; regular human insulin increased by 97.8 % and isophane insulin (NPH) by 78.0 %. Among the oral hypoglycemic agents, 5 mg glibenclamide increased by 65.9 %, and 500 and 850 mg metformin increased by 46.8 and 39.9 %, respectively, in the number of tablets dispensed in the first year of the SNTP Program. Among the hypoglycemic agents not available in SNTP, 4 mg glimepiride had the highest percentage increase in units supplied (19.2 %) in the same period. Among the insulin analogues, which were not available in the SNTP Program, insulin glulisine showed the greatest increase in units dispensed (34.2 %).

**Conclusions:**

The SNTP Program contributed to increased access to medicines for the treatment of diabetes in Brazil.

## Background

According to the World Health Organization (WHO), access to essential medications is one of the five indicators of progress in securing the right to health [1]. Although the percentage of the global population without access to essential medications has decreased, approximately 1.3–2.1 billion people still lack access to essential medications [2, 3]. In developing countries, especially in Latin America, larger differences in medication consumption levels exist between population strata [4]. Higher economic classes show consumption patterns similar to wealthier countries, while lower economic classes have difficulty in accessing basic medications [5].

According to Cameron et al. [6], even when medications are provided for free distribution or at low cost to the public sector, access is limited as a result of low availability. This low availability may result from a combination of factors such as inadequate funding, lack of incentives for stock maintenance, the inability to predict demand accurately, inefficient distribution systems or diversion of medications to the private sector.

Although the Brazilian Public Health System [Sistema Único de Saúde (SUS)] has the duty to provide medications for the treatment of diabetes that are included in the national list of essential medicines [7, 8], Bahia et al. [9] found that 24.6 % of patients with diabetes in Brazil bought their medications from private pharmacies.

To increase access to medications in Brazil, in 2006 the state began to subsidize approximately 90 % of the cost of drugs used for the treatment of diabetes and hypertension through a co-payment system associated with the Ministry of Health that covered medications purchased from private pharmacies [10]. In February 2011, this policy was expanded with the creation of the “Health Has No Price” Program [Programa Saúde Não Tem Preço (SNTP)], in which associated pharmacies began to provide two types of insulin (regular human insulin and isophane insulin or NPH) and three oral antidiabetic medications (glibenclamide 5 mg, metformin 500 and 850 mg) free of charge, with a subsequent reimbursement of the amount agreed upon between the government and the pharmaceutical industry [11]. In this context, the objective of our study was to evaluate the impact of the SNTP Program on the access to medications for diabetes treatment in Brazil.

## Methods

We performed a longitudinal observational study of the access to antidiabetic medications in Brazil after the implementation of the SNTP Program. Data were collected from 55,000 pharmacies, representing approximately 76 % of private pharmacy establishments in the country, from February 1, 2010 to January 31, 2012 [12]. The number of tablets (oral antidiabetic medication) and the international units (insulin and insulin analogues) supplied free of charge during the first 12 months of the SNTP Program were compared with those supplied under the co-payment system in the 12 months prior to the implementation of the program. The oral antidiabetic tablets selected were from two subgroups of the anatomical therapeutic chemical (ATC) WHO classification, which consisted of medicines in the SNTP Program (biguanides and sulfonylureas). In addition, an insulin subgroup was also included in the study. A similar analysis was performed with three fixed-dose combinations not included in the SNTP Program (two containing oral antidiabetics and one combined with insulin).

## Results

The number of supplied units of all medicines for the treatment of diabetes in the SNTP Program increased in the first 12 months of the SNTP Program compared with the 12 months prior to the implementation of the program. The percentage of variation ranged from 39.9 to 97.8 %. Four sulfonylureas (glibenclamide, glimepiride, glipizide and chlorpropamide), one biguanide (metformin) and three fixed-dose combinations marketed in Brazil were included in the analysis of oral antidiabetic medications. The number of tablets supplied in the SNTP program increased from 39.9 to 65.9 % in the first year of the program. No other oral antidiabetic medication showed a comparable increase in the number of units supplied. The number of tablets dispensed (none in the SNTP Program) of the three fixed-dose combinations analyzed decreased after implementation of the SNTP Program (Table 1).

**Table 1:** Number of oral antidiabetics tablets supplied in the first 12 months of the SNTP Program (free of charge) and in the 12 months before to the Program (copayment system)

Medicines	ATC	Feb. 2010–Jan. 2011	Feb. 2011–Jan. 2012	Percentage of charge
Medicines included in SNTP Program				
Glibenclamide 5 mg	A10BB01	158,388,380	262,747,850	65.9
Metformin 500 mg	A10BA02	289,027,638	424,146,878	46.8
Metformin 850 mg	A10BA02	283,321,454	396,338,216	39.9
Medicines not included in SNTP Program				
Single-drug formulations				
Glimepiride 4 mg	A10BB12	38,372,010	45,749,970	19.2
Metformin 1000 mg	A10BA02	12,940,380	14,202,110	9.8
Glimepiride 2 mg	A10BB12	63,221,310	67,166,190	6.2
Glipizide 5 mg	A10BB07	1,415,340	1,300,020	−8.2
Chlorpropamide 250 mg	A10BB02	17,151,540	14,763,550	−13.9
Fixed-dose combinations				
Glibenclamide + metformin (1 mg + 500 mg)^a^	A10BD02	25,764,510	22,236,330	−13.7
Glibenclamide + metformin (5 mg + 500 mg)^b^	A10BD02	41,330,990	36,197,800	−12.4
Glibenclamide + metformin (2,5 mg + 500 mg)^a^	A10BD02	12,895,200	11,573,430	−10.3

Brazil, February 2010 through January 2012

Adapted from information published [12]

*ATC* Anatomical therapeutic chemical

^a^ Only one of the combination of drugs is contemplated in the SNTP Program at the same concentration

^b^ Both drugs are included in the combination SNTP Program at the same concentrations

A similar evaluation was performed with insulin and the insulin analogues marketed in Brazil. The two types of insulins included in the SNTP Program showed the highest percentage of change in the number of international units dispensed, even when compared with the oral antidiabetics. The only form of insulin that showed a decrease in the number of international units supplied after the implementation of the program was the fixed-dose combination of regular human insulin and isophane insulin. Also, the insulin analogues not included in the SNTP Program (insulin lispro, glulisine, glargine, aspart and detemir) showed an increase in the number of international units dispensed (Table 2).

**Table 2:** Number of international units supplied in the first 12 months of the SNTP Program (free of charge) and in the 12 months before to the Program (copayment system)

Medicines	ATC	Feb. 2010–Jan. 2011	Feb. 2011–Jan. 2012	Percentage of charge
Medicines included in SNTP Program				
Regular human insulin	A10AB01	1,122,987	2,221,324	97.8
Isophane insulin (NPH)	A10AC01	11,435,131	20,352,054	78.0
Medicines not included in SNTP Program				
Single-drug formulations				
Insulin glulisine	A10AB06	429,221	575,883	34.2
Insulin detemir	A10AE05	1,644,945	1,864,980	13.4
Insulin lispro	A10AC04	1,278,932	1,396,643	9.2
Insulin aspart	A10AB05	1,554,705	1,696,280	9.1
Insulin glargine	A10AE04	3,328,109	3,533,336	9.1
Fixed-dose combinations				
Regular human insulin + isophane insulin (NPH)^a^	A10AD30	626,589	595,233	−5.0

Brazil, February 2010 through January 2012

Adapted from information published [12]

*ATC* Anatomical therapeutic chemical

^a^ Both drugs are included in the combination SNTP Program at the same concentrations

## Discussion

This is the first study describing the impact of the SNTP Program on the access to antidiabetics in Brazil. The results confirmed a significant expansion of access to the diabetes treatment medications included in the SNTP Program, especially compared with other medications of the same therapeutic classes that are available in the Brazilian market. The analysis of the percentage change in the number of oral antidiabetic tablets and the number of international units of insulin supplied, whether as single agents or fixed-dose combinations, indicates that access expansion was greater for medicines included in the SNTP Program.

It was further observed that a lack of some fixed-dose combinations in the SNTP Program may result in the prescription of two individual medicines. The four fixed-dose combinations analyzed and formulated with active ingredients of single agents can be obtained at no cost in associated pharmacies. These combinations exhibited a decrease in the number of units dispensed after the start of the SNTP Program. This result may indicate a preference for the prescription of medicines available through the program.

The implementation of the SNTP Program, through an agreement between private pharmacies and the Ministry of Health, increased the access to medicines for the treatment of diabetes in Brazil. Also, the number of associated pharmacies increased from 14,003 in December 2010 to 22,470 establishments in July 2013, covering 3664 cities [13, 14].

The initiative by the Ministry of Health to subsidize the purchase of medications in private pharmacies began in 2006 through a co-payment system in which the government was responsible for approximately 90 % of the cost of some antidiabetic and antihypertensive medicines. From 2011, the government began to pay the full cost of medications included in the SNTP program [9, 10]. The considerable increase in the number of antihypertensive medicines supplied during the implementation of the SNTP Program [14] confirms, once again, that the price of the medicine is an essential determinant of the economic accessibility and financial sustainability for individuals [15].

Mortality due to diabetes declined in Brazil over the 15 years between 1996 and 2011, minimally in men but considerably in women [16]. However, recent studies have shown a high prevalence of diabetes in Brazilian adults over time (11.9 %), with a progressive increase in the last 35 years [17]. It is estimated that hospitalization due to diabetes is responsible for 2.2 % of the Ministry of Health budget [18], and that 80 % of patients diagnosed with diabetes in the country (approximately 7.5 million) are assisted by SUS. Further, 57.4 % of patients with diabetes aged over 18 years received at least one medication for diabetes treatment in the Brazilian SNTP program in 2013 [18–20].

The number of dispensed units of oral antidiabetic medications included in the SNTP Program was eight times higher than the growth in the pharmaceutical market in Brazil during the same period (12.7 %). The highest percentage increase in oral antidiabetics was observed for 5 mg glibenclamide (65.9 %). The highest variation in the number of tablets was for 500 mg metformin, with an increase of over 135 million tablets compared with the 12 months preceding the implementation of the SNTP Program.

The insulin medications included in the SNTP Program showed the greatest significant changes in the number of international units dispensed. The increase was 97.8 % for regular human insulin and 78.0 % for isophane insulin (NPH). Among the antidiabetic medications not included in the SNTP program, only insulin glulisine significantly increased (34.2 %), but this increase was still less than the growth observed for the insulin types included in the program.

Despite the observed increase in the number of international units of insulin analogues dispensed, health technology assessments conducted by the National Technology Implementation Commission (Comissão Nacional de Incorporação de Tecnologias-Conitec) for SUS in 2013, recommended against the inclusion of the fast-acting insulin analogues (lispro, aspart and glulisine) for the treatment of type 1 diabetes [21] and the long-acting insulin analogues (detemir and glargine) for the treatment of types 1 and 2 diabetes patients [22].

It is important to note that a limitation of the results of this study did not include medicines used in hospital or dispensed for free at pharmacies of the Brazilian Public Health System, which according to Gialdi [23], represent approximately 30 % of medication consumption in Brazil. This study also did not address the economic aspects such as the amount reimbursed by the State to private pharmacies, which has been criticized for being much higher than the amount paid for the purchase of the same medications for distribution in the Brazilian Public Health System.

## Conclusions

The results of this study reveal that the SNTP Program has significantly contributed to an increase in access to medications for the treatment of diabetes, indicating that this public health policy has achieved its goal.

## References

[1] Hogerzeil HV, Mirta Z. Access to essential medicines as part of the right to health. In: World Health Organization, editor. The world medicines situation. 3rd edn. Geneva: World Health Organization; 2011.

[2] World Health Organization (2004). The world medicines situation.

[3] International Diabetes Federation (2013). IDF diabetes atlas.

[4] Rêgo ECL. Políticas de regulação do mercado de medicamentos: a experiência internacional. Revista do BNDES. 2000;7(14):367–400. http://www.bndes.gov.br/SiteBNDES/export/sites/default/bndes_pt/Galerias/Arquivos/conhecimento/revista/rev1414.pdf. Accessed 30 Apr 2015.

[5] Instituto de Pesquisa Econômica Aplicada. Programas de Assistência Farmacêutica do Governo Federal: evolução recente das compras diretas de medicamentos e primeiras evidências de sua eficiência, 2005 a 2008. Brasília: IPEA; 2010. http://www.ipea.gov.br/portal/images/stories/PDFs/comunicado/101216_comunicado74.pdf. Accessed 30 Apr 2015.

[6] Cameron A, Ewen M, Ross-Degnan D, Ball D, Laing R (2009). Medicine prices, availability, and affordability in 36 developing and middle-income countries: a secondary analysis. Lancet.

[7] Brasil. Presidência da República. Casa Civil. Lei nº 8.080 de 19 de setembro de 1990. Dispõe sobre as condições para a promoção, proteção e recuperação da saúde, a organização e o funcionamento dos serviços correspondentes e dá outras providências. Brasília: Subchefia para Assuntos Jurídicos; 1990. http://www.planalto.gov.br/ccivil_03/leis/l8080.htm. Accessed 30 Apr 2015.

[8] Giannella-neto D (2011). State of endocrinology and diabetology in Brazil. Indian J Endocrinol Metab.

[9] Bahia LR, Araujo DV, Schaan BD, Dib SA, Negrato CA, Leão MPS (2011). The costs of type 2 diabetes mellitus outpatient care in the Brazilian public health system. Value Health.

[10] Ministério da Saúde. Portaria GM/MS nº 491 de 9 de março de 2006. Dispõe sobre a expansão do Programa “Farmácia Popular do Brasil”. Diário Oficial da União, Brasília: MS; 2006. http://pesquisa.in.gov.br/imprensa/jsp/visualiza/index.jsp?jornal=1&pagina=59&data=10/03/2006. Accessed 30 Apr 2015.

[11] Ministério da Saúde. Portaria GM/MS nº 184 de 3 de fevereiro de 2011. Dispõe sobre o Programa “Farmácia Popular do Brasil”. Diário Oficial da União, Brasília: MS; 2011. http://pesquisa.in.gov.br/imprensa/jsp/visualiza/index.jsp?jornal=1&pagina=35&data=04/02/2011. Accessed 30 Apr 2015.

[12] Departamento de Treinamento e Desenvolvimento de Vendas. Tudo o que você precisa saber sobre: Programa Farmácia Popular do Brasil. Campinas: TeD Vendas EMS; 2013. http://pt.scribd.com/doc/241751804/EMS-REPORT-pdf. Accessed 30 Apr 2015.

[13] Ministério da Saúde. Portal da Saúde. Saúde Não Tem Preço: Acesso a medicamentos gratuitos cresce 273%. Brasília: MS; 2013. http://portalsaude.saude.gov.br/portalsaude/noticia/3942/162/acesso-a-medicamentos-gratuitos-cresce-273.html. Accessed 30 Apr 2015.

[14] Ministério da Saúde. Sala de Apoio à Gestão Estratégica do Ministério da Saúde: indicadores de saúde a um clique. Brasília: MS; 2013. http://189.28.128.178/sage/. Accessed 30 Apr 2015.

[15] Araujo JLO, Pereira MD, Fiol FSD, Barberato-Filho S (2014). Access to antihypertensive agentes in Brazil: evaluation of the Health has no price Program. Clin Ther.

[16] Schmidt MI (2015). Trends in mortality due to diabetes in Brazil, 1996–2011. Diabetol Metab Syndr.

[17] Cureau FV, Teló GH, de Souza MS, Côpes FS, Schaan BD (2015). Prevalence of diabetes mellitus in Brazil: a systematic review with meta-analysis Diabetol Metab Syndr..

[18] Pan American Health Organization. Access to high-cost medicines in the Americas: situation, challenges and perspectives. Washington: PAHO. 2010. http://apps.who.int/medicinedocs/documents/s19112en/s19112en.pdf. Accessed 30 Apr 2015.

[19] Rosa RS, Schmidt ML (2008). Diabetes mellitus: magnitude das hospitalizações na rede pública do Brasil, 1999–2001. Epidemiol Serv Saúde.

[20] Instituto Brasileiro de Geografia e Estatística. Pesquisa Nacional de Saúde 2013. Rio de Janeiro: IBGE; 2013. http://www.ibge.gov.br/home/estatistica/populacao/pns/2013. Accessed 10 Apr 2016.

[21] Brasil. Ministério da Saúde. Secretaria de Ciência, Tecnologia e Insumos Estratégicos. Departamento de Gestão e Incorporação de Tecnologias em Saúde. Insulinas análogas de longa ação diabetes mellitus tipo 2. Brasília:MS;2013. http://portalsaude.saude.gov.br/images/pdf/2014/janeiro/24/Relatorio-Insulina-diabetestipoII-CP.pdf. Accessed 30 Apr 2015.

[22] Brasil. Ministério da Saúde. Secretaria de Ciência, Tecnologia e Insumos Estratégicos. Departamento de Gestão e Incorporação de Tecnologias em Saúde. Insulinas análogas para diabetes mellitus tipo 1. Brasília:MS;2013. http://u.saude.gov.br/images/pdf/2014/fevereiro/28/Relatorio-Insulinas-analogas-diabetes-tipo-I-CP-114.pdf. Accessed 30 Apr 2015.

[23] Gialdi O. Access to high-cost medicines. In: Pan American Health Organization. Access to high-cost medicines in the Americas: situation, challenges and perspectives. Washington: PAHO. 2010. http://apps.who.int/medicinedocs/documents/s19112en/s19112en.pdf. Accessed 30 Apr 2015.

